# Factors associated with psychotropic drug use among community-dwelling older persons: A review of empirical studies

**DOI:** 10.1186/1472-6955-3-3

**Published:** 2004-08-13

**Authors:** Philippe Voyer, David Cohen, Sylvie Lauzon, Johanne Collin

**Affiliations:** 1 Faculty of nursing, Université Laval, Québec, Canada; 2 School of social work, College of health and urban affairs, Florida International University, Miami, USA; 3 School of nursing, University of Ottawa, Ottawa, Canada; 4 Faculty of pharmacy, Université de Montréal, Montréal, Canada

## Abstract

**Background:**

In the many descriptive studies on prescribed psychotropic drug use by community-dwelling older persons, several sociodemographic and other factors associated with drug use receive inconsistent support.

**Method:**

Empirical reports with data on at least benzodiazepine or antidepressant drug use in samples of older persons published between 1990 and 2001 (n = 32) were identified from major databases and analyzed to determine which factors are most frequently associated with psychotropic drug use in multivariate analyses. Methodological aspects were also examined.

**Results:**

Most reports used probability samples of users and non-users and employed cross-sectional designs. Among variables considered in 5 or more reports, race, proximity to health centers, medical consultations, sleep complaints, and health perception were virtually always associated to drug use. Gender, mental health, and physical health status were associated in about two-thirds of reports. Associations with age, marital status, medication coverage, socioeconomic status, and social support were usually not observed.

**Conclusions:**

The large variety of methods to operationalize drug use, mental health status, and social support probably affected the magnitude of observed relationships. Employing longitudinal designs and distinguishing short-term from long-term use, focusing on samples of drug users exclusively, defining drug use and drug classes more uniformly, and utilizing measures of psychological well-being rather than only of distress, might clarify the nature of observed associations and the direction of causality. Few studies tested specific hypotheses. Most studies focused on individual characteristics of respondents, neglecting the potential contribution of health care professionals to the phenomenon of psychotropic drug use among seniors.

## Background

The use of prescribed psychotropic drugs by older persons has been a subject of interest for several decades [1,2]. Psychotropic drugs are defined as substances that act directly on the central nervous system, affecting mood, cognition, and behavior, and usually include anxiolytics, sedatives and hypnotics, antidepressants, neuroleptics, anticonvulsants, and stimulants. The topic continues to draw researchers' attention for several reasons, including (1) the high prevalence of older users (especially of benzodiazepines) and their typically long-term consumption, (2) their special vulnerability to drug induced iatrogenesis, (3) the discrepancy between rates of mental disorder and rates of drug use among older people, and (4) inappropriate prescribing.

The prevalence of psychotropic drug use among community-dwelling older persons (usually defined as those 65 years and older) varies from about 20% to 48% [3-5]. More than half of them take psychotropics for six months or longer, and are therefore considered long-term users [4,6,7]. For example, 69% of Canadian elders using benzodiazepines have done so for at least a year [3]. Long-term use is contraindicated because benzodiazepines lack effectiveness beyond a few weeks or months of sustained use for their principal indications, the relief of insomnia and anxiety [8-17].

Since aging increases the likelihood of drug accumulation and intoxication, older persons are particularly vulnerable to adverse effects from psychotropic drugs [18]. Notable harmful consequences of psychotropic drug use include memory impairment, psychomotor slowing, delirium, falls with a risk of hip fracture, automobile accidents, and psychiatric hospitalizations [19-21].

Although more psychotropic drug users are found among older persons than any other age group, the prevalence of mental disorders appears to be lower among older than younger adults. This has been shown for major depression, anxiety disorders and sleep disorders [6,22-30]. Other observations, such as the small rate of hospital admissions for psychiatric reasons among older persons (1.1%) [4] compared to their high rate of psychotropic drug use relative to younger adults' rate, confirm these findings.

The inappropriate prescription of psychotropic drugs may account partially for the discrepancy between low levels of distress and high levels of drug use among older persons. Inappropriate prescriptions include questionable drug combinations (such as two benzodiazepines), excessive treatment duration, and drugs contraindicated for use by older people (such as long half-life benzodiazepines). Authors estimate that one-fifth to one-half of psychotropic drug prescriptions to the elderly are "inappropriate" [4,16,31,32].

These findings suggest that older persons' psychological well-being is not the principal determinant of their psychotropic drug use. In the present article, we review recent empirical studies in order to identify other factors that may account for psychotropic drug use among older persons. A large body of work bears on this age group and their use of psychotropic and other drugs [33,34]. Earlier reviews examined prevalence rates [35-39], the issues of long-term use [40] and dependence [41,42], as well as various models of drug use [43,44]. However, to our knowledge none focuses on community-dwelling psychotropic drug users. Consequently, the present review critically examines various factors associated with such drug use among this population. Each factor is discussed in terms of the empirical support it has received, of hypotheses put forth to explain its association with drug use, and of suggestions for future research.

## Methods

Reports were selected on April 20, 2001, from the following bibliographic databases: *MedLine, Cumulative Index to Nursing & Allied Health Literature*, *Psychlit*, *Eric *and *Sociological Abstracts *for the years 1990 to 2001. Various keywords were used. Selected reports had to: (1)constitute either a peer-reviewed journal article or a government publication published in English or French; (2) present specific retrievable empirical results concerning older persons, regardless of other age groups studied (some reports [16,39,45] were rejected because of this criterion); (3) report on non-institutionalized, community-dwelling participants at the time of the study; and (4) provide specific results on at least benzodiazepine or antidepressant drug use, regardless of other drug classes studied (some reports [29,46,47] were rejected because of this criterion). A total of 61citations met these criteria in the databases. Eliminating overlaps left only 32 separate reports, all of which were retrieved and are included in this review. Their reference lists were also consulted, but no other study was identified by this mean.

The 32 publications report on 30 different studies conducted in ten different countries. Unless indicated otherwise, each report is treated as a separate study. Most reports originated from the United States (31%) and Canada (28%), followed by France (12.5%), Sweden (9.5%), and 3% each (one report) from Australia, Austria, Ireland, the Netherlands, Spain, and the United Kingdom (Table 1 [see additional file 1]). Of 26 reports that disclosed a source of funding, 16 (61.5%) identified government agencies, 6 (23%) government-industry or government-foundation partnerships, and 4 (15.5%) industry. Most studies (62%) collected their data during the 1990s, but several did so during the 1980s (34%) and 1970s (3%). As far as could be determined, the median time from end of data collection to publication was 6 years (range: 1 – 12 years).

Most reports (24, 75%) presented multivariate data analyses, usually multivariate logistic regression, where the independent contribution of various variables to the variance in psychotropic drug use could be ascertained while controlling for the contribution of other variables. Although variables with statistically significant associations (p < .05) to drug use that remained in the regression equation were often considered "predictors," strictly speaking only an association is demonstrated in this fashion, and the direction of causality can rarely be established. This is especially so when a theoretical model is not specified prior to the data analysis, as was the case in the vast majority of reports. The eight remaining studies conducted bivariate analyses solely, and most examined three or less variables.

In the following review, associations of sociodemographic factors and life conditions with psychotropic drug use are examined first. A factor was deemed well supported by the review studies when 70% of the results from all studies that considered this factor were statistically significant and pointed in the same direction. Second, methodological and conceptual issues relevant to the study of psychotropic drug use among the elderly, and salient in the reviewed studies, are discussed.

## Results

### Psychotropic drug use and sociodemographic characteristics

#### Prevalence of psychotropic drug use

Weighted average prevalence rates of psychotropic drug use were estimated from all reports having collected data from probability samples or entire populations (n = 22). In longitudinal studies, data from the last year of the study were used. Average prevalence of any psychotropic drug use from 9 studies was 29.0% (range 11.8% – 42.5%). For drugs identified in the reports as benzodiazepines, minor tranquilizers, anxiolytics, or sedative-hypnotics, the average prevalence in 13 studies was 21.5% (range 6% – 43.8%). In 11 studies with data on antidepressants, the average prevalence was 6.9% (range 2.3% – 14%). Finally, for neuroleptics, it was 3.1% (6 studies, range 1% to 6%).

#### Age

Older people are more likely than any other age group to use any type of medication [29]. However, it has been observed that medication use decreases in those over 75 years [4]. Some suggest that this occurs because: doctors exercise more caution when prescribing to the oldest old [48,49], survivors into advanced age are healthier and thus use fewer medications [4,50], the elderly face fewer stressful events [50]. The observation of a decline in psychotropic drug use with very advancing age is not universal [51]: Blazer et al. [6] and Mamdani et al.[49] found that it reaches its peak prevalence among those older than 85 years. As Table 2 [see additional file 2 ] shows, 22 studies carried out 23 tests of the association between age and drug use, but only 8 (35%) of the results showed an age-specific trend (5 found an increase and 3 a decrease). This low percentage prevents firm conclusions about any age trend in regard to psychotropic drug use among older people.

#### Gender

Over the past three decades, numerous hypotheses have been proposed to explain the higher prevalence of psychotropic drug use among women than men: women are more inclined to reveal their emotional problems to their doctor[35,36,52-55], to request prescriptions explicitly [36,54,55], to hold more positive views of psychotropic drugs [50]. Some authors have suggested that men prefer to use alcohol rather than prescribed drugs to deal with emotional problems [56,57]. Graham and colleagues [42] failed to support this last hypothesis in a sample of 826 older persons. Other authors suggest that since women live longer than men, they experience more effects of aging, losses and health problems, all of which increase their likelihood of using a psychotropic [37,49,55,58]. Physicians might be more willing to prescribe a psychotropic drug to a woman than to a man [49,54,55,59]. Also, women visit physicians more often, increasing their chance of receiving a prescription [36,55,57]. However, in studies using population-wide databases, Brown et al.[60], Jorm et al.[55], and Weyerer and Dilling [45] controlled both the number of health problems and physician visits and showed that women still used more psychotropic drugs than men. Kirby et al. [53] found the same result when controlling for mental health status. Contradictory results are reported by Mayer-Oakes et al., [61] and Swartz et al., [62] who showed the use of benzodiazepines to be associated not to gender but to physical health status, poorer in women than in men.

In this review, 73% of the relevant studies support the established finding that women are more likely to use psychotropic drugs than men. In two studies, the relationship with gender holds only among those aged between 65 and 74 years, and in a third, the results are opposite. However, the studies shed little light on reasons for this disparity.

Only two studies identified as a secondary objective to look at the gender issue in relation to psychotropic drug use [42,49]. A few studies tested hypotheses to examine the role of physical health status and mental health status relative to gender among seniors, but none received consistent support [42,45,53,55,60-62]. No other hypothesis was directly tested in this body of studies. Some authors borrowed hypotheses from studies with middle-aged adults to discuss their results [49,53,55,63]. Authors in other studies which found a statistically significant relationship between gender and psychotropic drug use did not discuss or interpret the association [6,48,55,60,64-74]. In sum, many studies confirm that more women than men use psychotropic drugs, but no single compelling explanation for this difference among the aged emerges from this body of studies.

#### Race

The role of culture, ethnicity, and race has received little attention so far in the literature on older persons' psychotropic drug use. Explaining cultural, ethnic, or racial disparities in use of health services is a complex endeavor, requiring analysis of predisposing, structural, access, and other variables in interaction [75-78]. In this review, two studies from Canada compared likelihood of use among French and English speakers, one finding an association with French speakers, albeit in a non-probability sample [79]. In addition, six studies from the United States compared the prevalence rate of psychotropic drug use between Whites and African-Americans. All studies but one controlled for income or education, and all found that Whites are significantly more likely to use psychotropic drugs. These results concur with many findings showing differential use of health services along racial and ethnic lines in the United States [75].

Interpretations of the race-specific findings from these six studies focused mostly on professional-and individual-level determinants. Brown et al. [60] suggested that doctors prescribe fewer psychotropic drugs to older African-Americans by prudence, since the former are more sensitive to some drug effects. Other authors proposed that different prescription patterns result from differences in the expression of psychological distress [62,80] or from the lower rate of depression among African-Americans [6]. The smaller proportion of African-Americans and other minorities in the United States with private health insurance also may play a role, especially with regard to newer, more expensive drugs [81]. In that country, more studies are needed to test these hypotheses and others involving structural and access variables, also with other significant minority groups such as Latinos. Beyond racial or ethnic status, studies are needed to understand whether distinct cultural attitudes independently predict psychotropic drug use among older people after various sociodemographic and structural variables are controlled.

#### Marital status

It has been suggested that older widows would be more likely to use psychotropic drugs because of the distress of bereavement [82]. Eleven of 32 studies examined psychotropic drug use in relation to marital status, but only 4 (36%) confirmed an association between drug use and not being married (single, widowed, divorced, or separated). A spouse's death, especially if preceded by long illness, is likely to represent a significant stressful event accompanied by insomnia, anxiety or depression for any person and this can lead to psychotropic drug use. However, strictly speaking, stressful events or psychological distress rather than marital status as such would be implicated, and perhaps only on a relatively short-term basis. In summary, marital status so far is not revealed as a significant factor accounting for psychotropic drug use in older people.

#### Socioeconomic status

Living in deprived environments and having an unskilled occupation, low income and little formal education are variables associated with psychological distress among adults, and it might be assumed that the same would hold among older people. However, the studies reviewed fail to support this association. Socioeconomic status (SES) is typically operationalized by education, income, and occupation. As Table 2 [see additional file 1 ] shows, 13 studies examined one of more of these variables. Lower education was significantly associated with drug use in only 3 of 11 results (27%). Lower income was significantly associated with drug use in 3 of 7 results (43%). Two studies examining occupational status before retirement found an association: one found that the self-employed were less likely to use psychotropics [83], the other, using a non-probability sample, found more blue-collar workers among users [79]. In sum, most studies did not support the impact of lower educational level or lower income on psychotropic drug use among older persons. The sparse findings regarding occupation are difficult to interpret.

While the importance of SES as a predisposing or mediating influence on drug use might be more established in general population studies, it may be less relevant in the older age group because of a lower rate of psychological distress and greater access to income security plans and retirement pensions that prevent extreme poverty.

#### Insurance status

In the United States and until recently in Canada, older persons benefited from Medicare or provincial health insurance plans without medication coverage. Insurance coverage has been shown to influence psychotropic drug use by the elderly [84]. Five studies in this review (four from the USA and one from Canada) examined this issue in six different tests but only two (33%) supported a relationship between having insurance coverage for medication and being more likely to use psychotropic drugs. Besides the fact that the most frequent psychotropic drugs used by older persons during the time period covered by the 32 studies in this review (for example, benzodiazepines like lorazepam or tricyclic antidepressants) were available in inexpensive generic versions, no compelling hypothesis exists to account for these results. However, newer psychotropics, such as selective serotonin reuptake inhibitors which reached peak usage during the late 1990s, have been marketed at much higher prices, and the impact of insurance coverage on their use might be shown to differ in future studies.

#### Proximity to health centers

No study examined meso-level variables related to SES, such as census-tract or neighborhood poverty, except proximity to health centers. The use of health services, including medications, may be increased by proximity to such services [85]. Close or easy availability of health services augments medical consultations (and consequently the request of medication or its prescription). This hypothesis is supported in six of seven studies (85%) in the present review.

### Life conditions

#### Stressful events

Stressful events may increase people's likelihood of using psychotropic drugs. Only three studies examined this relationship, each reporting a different result: a positive association [65], a negative association [50], and no association [79]. The precise role of stressful events within a model of psychotropic drug use by older persons clearly needs to be elucidated. To explain the mixed results, future research might consider the transformation of stressors over time: stressful events may impact significantly on the initiation of psychotropic drug use, but may recede to a negligible level in long-term consumption. A longitudinal design is needed to examine this relationship more adequately, but unfortunately none of the 12 longitudinal studies in this review explored stressful events in relation to psychotropic drug use.

#### Illnesses and other medications

A recent qualitative synthesis of the literature concludes that the pathway to psychotropic drug use among older people typically includes the presence of organic disease [86] (and loneliness, see ahead). Since elderly people suffer from more diseases than younger people, they might use more psychotropic and of course non-psychotropic medications. In another vein, some anxious elderly users of psychotropic drugs might worry excessively about their health and be more likely to consult physicians and receive other medications [85].

In this review, nine of 16 studies (56%) found a significant association between the presence or the number of physical illnesses and psychotropic drug use. In their conceptual model, Gustaffson et al. [69] suggest that affliction by a new disease constitutes a stressful event that worsens an older person's mental health status, leading to the need (personally or professionally perceived) for a psychotropic drug. Nonetheless, the results overall are equivocal, perhaps because researchers neglected to consider both the type and the duration of health problems. For example, living with high blood pressure and being struck with congestive heart failure are dissimilar experiences. Typically, researchers counted only the presence or number of illnesses and did not distinguish between individuals who have been living with a disease for a long period of time and those recently experiencing it. As actually measured, the illness factor did not clearly discriminate between older users and non-users of psychotropic drugs. Taking into account both the nature and the duration of illnesses in future studies might better elucidate the relationship between health status and psychotropic drug use.

Four of six studies (60%) examining the relationship between psychotropic drug use and use of other medications found a significantly positive association, but the direction of causality remains unclear.

#### Health perception

Health perception–the self-evaluation of one own's health–has been shown to be more strongly associated with psychotropic drug use than actual diagnosis of disease [33]. Some researchers see here an indirect relationship, where poor health perception negatively influences the mental health status of a person, which in turn leads to the request for psychotropic drugs [65,69]. Previous studies support the correlation between mental health status and health perception [87-90].

In this review, seven of eight studies (87.5%) examining the association between health perception and drug use found a positive relationship. However, two thirds of the reviewed studies were cross-sectional, preventing conclusions about cause-effect relationship, and typically more than 80% of the psychotropic drugs used by their subjects were benzodiazepines. This raises the possibility that some subjects had poor health perception as a result of benzodiazepine consumption. Iatrogenic effects of long term benzodiazepine use may worsen health and functional capacity, hence health perception [73,91]. The present findings confirm that health perception has a place within a model of psychotropic drug use among the elderly, yet leave its precise role unclear. A longitudinal study would help to elucidate this role.

#### Social support

It has previously been proposed that low social support is associated with psychotropic drug use among older people [92,93]. In 10 studies here reviewed, various scales operationalized constructs identified as social support, social network, social relationships, family relationships, and social isolation. A variable of living alone or with others was included in two of these studies, and subjective reports of loneliness in two studies. However, in 13 tests of the association between some of these variables and drug use, 9 results (69%) failed to observe any significant relationship. All five tests of association with "social support" and all three tests of association with "social relationships" found no relationship with drug use. The single association of "social relationships" was positive, as was one of two of "living alone." Overall, results are counter-intuitive since one would expect that without significant social support to aid in time of difficulties, a person might be more likely to seek medical help, increasing the chances of receiving a drug prescription.

Either social support is a minor strand in the tapestry of psychotropic drug use, or the concept of social support may be inadequately operationalized by standard scales. These may need to measure, beyond frequency of social contacts and residential status, subjective judgments of loneliness and of opportunities for social intimacy. Whereas a summed scale score indicating "social isolation" was not associated with benzodiazepine use in one study [61], in both studies where the older person's feeling of "loneliness" as such was elicited, its association with psychotropic drug use was found to be significant after controlling for other variables, including "social support" scores [69,70]. Rather than the presence or extent of social support, a subjective feeling of loneliness may influence, directly or indirectly, psychotropic drug use [86].

#### Mental health status

The association between a diagnosis or rating of mental disorder or psychological distress and the use of psychotropic drugs is evidently anticipated. In 18 studies, 22 tests of this association were carried out, and it was significant in 17 (77%). An intriguing observation is that two of the four studies that failed to support the association included only the antidepressant drug class [67,71]. While the association between mental health and psychotropic drug consumption is well supported, it is worth noting that the magnitude of the association varies considerably across studies. The following two pairs of longitudinal and cross-sectional large-sample studies using multivariate logistic regression analysis illustrate this variability: benzodiazepine users were 2.13 times more likely than non-users to report depressive symptoms at 10-year follow-up [6], and 6.7 more likely to report emotional or nervous problems [63]. Psychotropic drug users were 2.78 times more likely to report depressive symptoms at 6-year follow-up [68], and 4 times more likely to report anxious or depressive symptoms [94] (Table 3). We discuss ahead how this variability may stem from methodological differences, especially in the measurement of the key variables in the associations.

**Table 3:** The association between mental health variables and psychotropic drug use

**Study**	Results
^65^Allard et al. (1995), n = 500	In bivariate analysis, drug use negatively correlated with morale (r = -0.32, p < 0.001)
^6 ^Blazer et al. (2000), n = 4,162	In multivariate logistic regression, benzodiazepine use significantly associated with depressive symptoms at 10-year follow-up (OR: 2.13, p < 0.05)
^68 ^Dealberto et al. (1997), n = 2,812	In multivariate logistic regression, drug use significantly associated with depressive symptoms at 6-year follow-up (OR: 2.78, p < 0.001)
^69 ^Gustafsson et al. (1996), n = 421	In LISREL model, drug use positively correlated (0.63) with poor mental health (chi^2 ^= 3.38, p = 0.33)
^94 ^Paterniti et al. (1998), n = 1,389	In multivariate logistic regression, drug users 4 times more likely to report anxious and depressive symptoms than non-users (OR: 4.0, CI: 2.5–6.5)
^63 ^Gleason et al. (1998), n = 5,181	In multivariate logistic regression, benzodiazepine users 7 times more likely to report emotional or nervous problems than non-users (OR: 6.66, CI 5.1, 8.7)
^74 ^Taylor et al. (1998), n = 5,222	Depressive symptoms multiply by 3 the likelihood of using hypnotics and by 5.1 the likelihood of using anxiolytics. Anxious symptoms multiply by 4.2 the likelihood of using hypnotics and by 3 of using anxiolytics.

#### Sleep

In the general population, Ohayon and Caulet [28] have clearly shown that a sleep disorder, such as primary insomnia diagnosed according to *DSM-III-R *criteria [95], is associated with the use of psychotropic drugs. These authors also noted that complaints of poor or inadequate sleep were associated with drug use. In the present review, all five studies (100%) that evaluated this latter association among older persons supported it. Although as mentioned earlier they do not report more sleep problems than younger persons, the presence of sleep complaints appears to play an important role in their psychotropic drug use.

#### Medical consultations

The number of annual visits to a doctor's office has an obvious influence on the number of prescriptions since it has been shown that up to 75% of visits by older people end up with a prescription [96,97]. All seven studies (100%) having examined this factor in this review supported its association with psychotropic drug use. Because no drug can be prescribed without the active participation of a physician, the physician's role is undoubtedly critical. However, it might differ over time. It has been previously suggested [98] that the first prescription of a benzodiazepine drug and the renewal prescription are two separate phenomena: the doctor might be more directive during the visit leading to the first prescription, whereas the older patient might be more directive to ensure its renewal. Thus, the physician's role in psychotropic drug use among community-dwelling older persons might best be modelled according to the duration of use. In previous research, several characteristics of physicians, notably a high number of consultations, have been significantly associated with the likelihood of prescribing psychotropics to older patients [99].

#### Summary of findings on sociodemographic factors and life conditions

Among the 16 factors identified in this review of 32 empirical reports, only the variables of language and stressful events were examined in less than five different studies. Gender, age, and mental health status were most frequently examined. The variables of race, medical consultations, proximity to health centers, sleep complaints and health perception are significantly associated with drug use in all or almost all studies incorporating them. Gender and mental health status are associated with drug use in over 70% of studies, while physical illness and number of medications are associated in slightly over half of relevant studies. However, significant associations with age, marital status, socioeconomic status, and social support are observed in only 27% to 36% of studies in which these factors are examined.

### Methodological issues in the study of psychotropic drug use by the elderly

Conflicting results could be explained partially by methodological and conceptual characteristics of many of the studies reviewed. The following discussion focuses on study design, sample, concept definitions and measurements (particularly of mental health status and psychotropic drug use), as well as distinction between short-and long-term psychotropic drug use. Table 1 [see additional file 1 ] lists these and other methodological characteristics of the studies reviewed, as well as summaries of findings on prevalence of psychotropic drug use.

#### Design

Descriptive methods (100%) using cross-sectional data (62%) predominate in this body of recent reports of psychotropic drug use in the elderly. Cross-sectional data are vulnerable to sample bias, especially when respondents are not randomly selected, as was the case in 9 of 30 (30%) studies. Given the greater costs involved, the proportion of longitudinal research (38%) seems respectable. Possibly because of cost factors, these studies examined fewer variables in relation to psychotropic drug use. However, besides highlighting the phenomenon of long-term use of psychotropic drugs by the elderly [55], they provided clear demonstrations of the deleterious effect of psychotropic drug consumption on cognitive capacities [19] and of how nursing home admission increases psychotropic drug prescriptions [5], for example. Despite their greater cost and demanding logistics, longitudinal designs are essential to understand more accurately how and why an older person begins, ceases, continues, and modulates psychotropic drug use. Finally, the absence of qualitative studies, which usually allow for a better grasp of elderly users' own perspectives on their use, is noteworthy; most studies so far have reflected only the researchers' points of view.

#### Samples

Virtually all samples (29 in the 30 distinct studies) were composed of older consumers and non-consumers of psychotropic drugs. Comparisons between these two groups allow researchers to identify what differentiates users from non-users of psychotropic drugs. However, one notices from this review that researchers do not always succeed. One possible reason may be that short-and long-term users are combined in one group. Indeed, in almost every study it appears that investigators are comparing long-term users with non users. About 20% to 30% of community-dwelling older persons use psychotropic drugs [3,84], with over two-thirds having been users for more than a year, and over one half for more than one year. Among the very old, up to 93% have consumed for more than a year [100]. Thus, the characteristics of users with less than six months of use are not well known. In one longitudinal study, the one factor that most accurately distinguished long-term users of psychotropic drugs from non-users was having been a user of the drug at the first measurement three years earlier [68]. The researchers found that older users were 15 times more likely to be users 3 years later – 71 times more likely in the case of antidepressant drugs – whereas the fact of being depressed increased the likelihood of drug use at follow-up by 4.7 times.

Thus, if most psychotropic drug use among the elderly transforms into long-term use, the process of transformation itself requires more observation and explanation, again by means of longitudinal research. Immediate problems leading to the first prescription of a psychotropic drug, such as psychological distress or insomnia, might have little relevance among long-term users. Possibly, drug dependence has developed among some long-term users and sustains long-term consumption [41]. The six-month cut-off period habitually used to distinguish short-from long-term consumption in studies may be too long if dependence is taken into account, as patterns of physiological and psychological dependence may already have become established within such a time frame. However, current studies have not taken into account the dependence issue. For instance, one can wonder how withdrawal symptoms are influencing beginning and long-term users of psychotropic drugs. Fear of withdrawal symptoms might explain long-term use of a certain percentage of elderly users. In one previous study, 71% of middle-aged psychotropic drug users wanted to stop consuming these medications, but half of them feared stopping because of withdrawal symptoms [47]. It seems desirable to increase knowledge on this aspect of the phenomenon among older people, given the well documented potential of benzodiazepines to provoke dependence.

It also appears that over the past decade, the use of mixed samples of users and non-users of psychotropic drugs has provided little information about older consumers themselves. Although comparison is essential for understanding, perhaps more studies should target older drug users exclusively. For instance, we know of no study examining mental health outcomes of older psychotropic drugs users over time. One might deem it illogical if no studies had been conducted on the long-term effects of antihypertensive medication on blood pressure. Grad [101] made the same observation when commenting on the few empirical studies of the impact of long-term use of benzodiazepines on the quality of sleep of community-dwelling older persons.

#### Mental health status, distress, and psychological well-being

The concepts of mental health, psychological distress, depression, and social support are often studied in relation to psychotropic drug use, but operationalized differently across studies. For instance, in the body of studies here reviewed, mental or emotional state is generally measured by standardized, self-rated scales that focus on the manifestation or experience of various symptoms of psychological or bodily distress, or by structured diagnostic interviews aiming to identify mental disorders according to official diagnostic criteria. In all, at least 12 different instruments or scales, 4 different systems of diagnostic criteria (ICD-9 and ICD-10, DSM-III-R and DSM-IV), and 3 unstructured open questions were used in 18 studies to measure concepts identified as depression (12 studies), anxiety (6 studies), psychological distress, mental disorders, life events (3 studies each), psychiatric syndromes (2 studies each), and morale, nervous or emotional disorders, melancholy, nerves, emotional condition, dementia, and sleep problems (1 study each). This variability in concepts and instruments surely impacts the variability of observed relationships between drug use and mental health status (Table 3).

In addition, it is relevant to ask whether the sole use of scales measuring psychiatric symptoms is appropriate to understand psychotropic drugs use among older people. As discussed, the rate of mental disorders, including sleep problems, is lower among older than middle-aged adults, and largely outpaces their use of psychotropic medications. This suggests that many older people are prescribed psychotropic drugs for mild to moderate psychological difficulties that would not qualify as DSM mental disorders, and that might not produce significant impairment in daily functioning. For example, in two studies [61,79], while drug users displayed more depressive symptoms than non-users according to the Center for Epidemiologic Studies Depression scale (CES-D), their scores did not reach the standard threshold of 16 points necessary for a diagnosis of depression. Results such as these suggest that scales less oriented to symptoms, distress, or disorder, such as measures of psychological well being that focus on so-called "positive" psychological dimensions such as self-acceptance, autonomy, relationships, and purpose in life [102-104], should supplement traditional-type scales. Psychological well-being is not merely the reverse of psychological distress, and the relationship of psychological well-being to symptoms of distress is complex [105]. No scale of psychological well-being was used in any of the reviewed reports. However, in one recent study of older persons, Guerette [106] found that among several measures of mental status, measures of psychological well-being produced the strongest associations with benzodiazepine use.

#### Measures of psychotropic drug use

Some conflicting or ambiguous results across studies can be attributed to how psychotropic drug use should be measured. Two issues are discussed here: what drug classes to include, and what time period of use to consider.

Depending on the study, one or more different classes of drugs are included among the substances measured as psychotropic medications, although, for example, indications for the prescription of benzodiazepines differ from those for the prescription of neuroleptics. Using a composite variable of any psychotropic drug use when examining the association with relevant sociodemographic factors or life conditions probably affects the observed association and consequently our understanding of the phenomenon. It would seem better to determine separately what variables are associated with benzodiazepine, antidepressant, and other drug use. Benzodiazepines warrant careful attention, as these drugs may be classified as anxiolytics, sedatives, hypnotics, and anticonvulsants–and we would not expect logically the correlates of anticonvulsant use in the elderly to resemble those of hypnotic use. In addition, researchers rarely specify which drugs they classify as "minor tranquilizers," or how they distinguish between "anxiolytics" and "sedatives." These definitional issues have long existed in pharmacoepidemiology and we should not expect them to be solved in these studies, but without assurances that drug categories do not overlap, measures of the dependent variable occasionally remain imprecise.

The average prevalence of neuroleptic drug use among community-dwelling older persons, estimated at 3.1% from six studies with probability samples, deserves comment in this respect. It has been common wisdom that such drug use has been rare except in institutionalized samples. The studies also confirm that the main psychotropic drugs prescribed to seniors are benzodiazepines and antidepressants. However, most of these studies, conducted in the early 1990s, probably missed the increased popularity of atypical neuroleptics. These drugs have so far enjoyed a reputation as less toxic drugs than conventional neuroleptics, encouraging their prescription among the older age group [98]. Several problems associated with benzodiazepine use among seniors are not associated with neuroleptics. However, all neuroleptics carry their own substantial risks of adverse effects, such as tardive dyskinesia and cognitive dysfunction (conventionals) and weight gain and diabetes (atypicals) [107,108]. In future studies, investigators will probably need to examine the prevalence of these drugs' use among the elderly. The category of reversible cholinesterase inhibitors, also known as anti-dementia, anti-Alzheimer's, or "cognition enhancers," is also unmentioned in any of the reviewed studies. Introduced in the mid-1990s, drugs such as donazepil and rivastigmine are increasingly prescribed to community-dwelling elderly showing subtle signs of dementia and simple forgetfulness (often termed "mild cognitive decline") according to a preventive ethos [109].

The second measurement issue to consider relates to the period of time for which data are collected, a source of confound noted by other reviewers [41,42,110,111]. In the 30 studies, temporal windows varied widely, from "current use" (4 studies), "current and past use" (1 study), "regular use" (2 studies), two days (2 studies), one week (1 study) two weeks (3 studies), one month (3 studies), three months (5 studies), and one year (7 studies). In two studies this period is undefined. When drug utilization is measured based on more than a week, one risks inflating the prevalence rate by including consumers who no longer use the drugs. Conversely, one risks omitting occasional users when referring to the last two days only [45]. Graham and Vidal-Zeballos [42] suggested asking respondents about short-term consumption (past 2 days and "recent use") and about long-term use (past year). This suggestion does not however solve problems of inaccurate self-report. Studies have found that older persons frequently hide or deny their use of psychotropic drugs [112-114].

To avoid these pitfalls, researchers in six studies determined psychotropic drug use according to municipal, provincial, state or national prescription databases held by government agencies or health maintenance organizations. The number of prescriptions, however, can greatly differ from actual drug consumption [16]. For example, about 50% of the elderly do not comply with their prescription regimen [115,116]. and an unknown proportion share their pills with relatives or friends [85].

Finally, researchers in at least 10 studies measured drug use by inspecting medication containers at the home of the respondent [83]. This method confirms that drugs have been obtained but leaves the compliance problem unresolved. A good rationale exists nonetheless to encourage this method, probably the most reliable way to get as close as possible to actual consumption [55,63,83,111]. Given that one third of studies in this review used this method suggests that its cost may be relatively acceptable. Interestingly, only two studies used telephone surveys. Owing to their relatively lesser cost than personal interviews, and since the development of reliable technologies, telephone surveys using random-digit dialing and random-select dialing are increasingly used to contact respondents in health related surveys.

## Conclusions

Without being exhaustive, the present literature review is comprehensive and the range of reports, methods, samples, and geographical locations provides a reasonably solid base to support most recommendations. This review was limited to empirical studies. To better understand the phenomenon of psychotropic drug use among community-dwelling elderly, it is desirable that historical, psychological, sociological, political and regulatory, as well as medical aspects of the phenomenon be considered.

While some factors are clearly associated with psychotropic drug use in the reviewed studies (such as race, proximity to health centers, sleep complaints, and health perception), few investigators test specific hypotheses to account for the associations. For example, sparse work focuses on cultural factors that might explain drug use disparities between Whites and African-Americans. Similarly, with most drug users being long-term and most drug treatments for insomnia losing their effectiveness with long-term use, the strong association between sleep complaints and drug use needs thorough examination.

One of the least studied aspects of the phenomena in the reviewed studies concerns the role of health care professionals. Physicians, nurses, and pharmacists all interact significantly with community-dwelling older persons, especially with those who take psychotropic medications. Only through a health care professional such as a physician and a pharmacist would the vast majority of older people obtain a psychotropic drug to relieve a sleep problem. Recently, the Internet and mail-order pharmacies without face-to-face interaction have facilitated individuals' access to prescription drugs. Each professional's role, as mentioned, is also likely to alter as the individual's phase of consumption transforms from short-to long-term. Researchers must face the challenge to incorporate variables related to health care professionals' attitudes and behaviors, as well as to new modes of distribution of psychotropic drugs to consumers, in their study of the phenomenon.

Approximately one third of community-dwelling older persons use psychotropic medications. If the rate of psychiatric disorders among a population serves as a guideline, then obviously older persons' use of psychiatric drugs far outpaces these drugs' standard indications and has extended into areas where drugs have little documented effectiveness. Viewed in this light, the ubiquitous phenomenon of long-term psychotropic drug use should evoke concern and caution. The discipline of nursing can definitely contribute to the rational use of these agents among older people. As suggested in this review, researchers do not still fully grasp the dynamics of psychotropic drug use among this population and creative and rigorous research from several disciplines, and from interdisciplinary perspectives, is needed. However, nurses concerned by the problem of the overuse of medication and its adverse consequences can already implement and evaluate programs to educate older people and allied health care and social service professionals about the risks of psychotropic drugs and alternatives to drugs for the management of everyday anxiety, loneliness, depression, and especially insomnia. Nurses can also actively implement and evaluate drug withdrawal programs aimed at long-term users who have had difficulty in withdrawing, and especially at short-term users who might soon be trapped into dependency and thus long-term use. Conversely, diverse patterns of psychotropic drug use undoubtedly exist among older persons, and positive patterns of use, emanating from users' own experiences and discoveries, need to be documented and disseminated.

## Competing interests

None declared.

## Authors' contributions

PV and DC conducted the literature review and drafted the manuscript. SL and JC revised the literature review and subsequent drafts. All authors read and approved the final manuscript.

## Pre-publication history

The pre-publication history for this paper can be accessed here:



## Supplementary Material

Additional File 1 Characteristics of 32 empirical reports on psychotropic drug use among community-dwelling older persons, 1990–2001. Reports on psychotropic drug use among community-dwelling older persons. Click here for file

Additional File 2 Factors associated with psychotropic drug use among community-dwelling older persons in 32 empirical reports, 1990–2001. Factors associated with psychotropic drug use among community-dwelling older persons. Click here for file
