# Supplementary material for: Factors associated with psychotropic drug use among community-dwelling older persons: A review of empirical studies
Source: BMC Nurs. 2004 Aug 13;3:3. doi: 10.1186/1472-6955-3-3 (PMC514897; doi:10.1186/1472-6955-3-3)
Supplement: Additional File 1 — Characteristics of 32 empirical reports on psychotropic drug use among community-dwelling older persons, 1990–2001. Reports on psychotropic drug use among community-dwelling older persons. [file 1472-6955-3-3-S1.doc]

# **Table 1**

Characteristics of 32 empirical reports on psychotropic drug use among

community-dwelling older persons, 1990-2001

| **Authors, Publication year** | **Country,**  **Study year(s)** | **Design^1^** | **Sample selection, Sample size^2^** | **Mental health measures** | **Data collection method,**  **Drug use period** | **Prevalence of psychotropic drug use** |
| --- | --- | --- | --- | --- | --- | --- |
| ^65^Allard et al., 1995 | Canada  1991 | C | P  500 | Philadelphia Geriatric Center  Morale Scale | Home interviews,  past 3 months | Any psychotropic: 31.8%  Minor tranquillizers: 20.8%  Hypnotics: 12.3%  Antidepressants: 4.0%  Antipsychotics: 2.0% |
| ^66^Antonijoan et al., 1990 | Spain  1988 | C | NP  126 | Symptom Distress Checklist  (SCL-90) | Agency interview,  past week | Any psychotropic: 16%  Benzodiazepines*:* 13.7%  Antidepressants: 2.2% |
| ^19^Berg & Dellasega, 1996 | Sweden  ~ 1979-88 | L | P  743 | - | Home and outpatient clinic interviews | Any psychotropic at 70, 75, & 79 yrs: 29%, 39%, & 36%  *- Anxiolytics & hypnotics: 90%* |
| ^6^Blazer et al., 2000^3^ | USA  1986-96 | L | P  4,000 | Center for Epidemiologic Studies Depression Scale (CES-D) | Home interview with containers, past 2 weeks | Any psychotropic 1986-96: 13.3 — 11.8%  Benzodiazepines: 12.0 — 10.2%  Non-BZD sedatives & hypnotics: 1.6 — 0.7% |
| ^67^Blazer et al., 2000b^3^ | USA  1986-96 | L | P  4,162 |  | Home interview with containers, past 2 weeks | Antidepressants 1986-96: 3.8 - 11% |
| ^60^Brown et al., 1995 | USA  1981-83 | C | P  13,074 | CES-D | Home interview with containers, past 2 weeks | Antidepressants in 4 states: 2.4 — 4.1% |
| ^119^Cans & Rotily,  1991 | France  1990 | C | P  257 | - | Telephone interview,  past 3 months | Any psychotropic: 37%  Benzodiazepines*:* 24.4%  Antidepressants: 4.1% |
| ^68^Dealberto et al., 1997 | USA  1982-88 | L | P  2,812 | CES-D | Home interview,  past 2 days | Any psychotropic 1982-88: 12.3 — 15.1%  Benzodiazepines: 6.25 — 6.0 %  Antidepressants: 2.0 — 4.3%  Anticonvulsants: 2.0 — 2.6%  Neuroleptics: 1.5 — 2.6% |
| ^48^Egberts et al., 1997 | Netherlands  1991-95 | L | Population  7,812  ≥ 55 yrs | - | Gov’t prescription dispensing database | 1-, 2-, & 3-year cumulative incidence of starting antidepressants: 1.3%, 2.7%, & 4% |
| ^63^Gleason et al., 1998 | USA  1989-90 | C | N-P  5,181 | “self-reported physician-diagnosed nervous or emotional disorder” | Home interview with containers,  past 2 weeks | Benzodiazepines: 9.9% |
| ^42^Graham et al., 1998 | Canada  1990 | C | P  826 | - | Home interview with containers,  past 4 weeks | Tranquillizers and/or sleeping pills: 20.6% women, 14.3% men |
| ^69^Gustafsson et al., 1996 | Sweden  1986 | C | P  421  ≥75 yrs | Self-reports of “insomnia, melancholy, or anxiety” | Gov’t prescription dispensing database | Any psychotropic: 37%  Hypnotics/sedatives/anxiolytics: 33%  Neuroleptics: 6%  Antidepressants: 4% |

| ^55^Jorm et al., 2000 | Australia  1991-95 | L | P  337 | CES-D,  “nerves or an emotional condition for which you are receiving treatment” | Home interview with containers, regular use | Benzodiazepines: 16.6% using continuously 1991-95 |
| --- | --- | --- | --- | --- | --- | --- |
| ^53^Kirby et al., 1999 | Ireland  1993-97 | C | P  1,701 | Psychiatric syndromes: Geriatric Mental State (GMS-AGECAT) | Home interview with containers, current use | Any psychotropic: 21.9%  Benzodiazepines: 17.3%  Antidepressants: 5%  Neuroleptics: 1.3% |
| ^70^Larose, 1996 | Canada  1994 | C | P  549 | Geriatric Depression Scale (GDS) | Home interview with containers, current use | Minor tranquillizers (64.3 %)  Major tranquillizers (1 %)  Hypnotics (23.4 %)  Antidepressants (12.3 %) |
| ^49^Mamdani et al., 1999 | Canada,  1993-97 | L | Population  1.4 million | - | Gov’t prescription claims database | Antidepressants: low of 5.6% in 65-69 yr-old men in 1993 to high of 17.2% in 85-89 yr-old women in  1997 |
| ^61^Mayer-Oakes et al., 1993 | USA  ~ 1989 | C | N-P  1,752 | CES-D,  Functional Status Questionaire (FSQ) | Mailed survey, at least twice in past 12 months | Benzodiazepines: 20% |
| ^64^McNutt et al., 1994 | USA  1989 | L, 1 yr | N-P  20,944 | - | Gov’t benzodiazepine prescription database | 1^st^ quarter 1988 — 3^rd^ quarter 1989:  Benzodiazepines: 29% — 15%  Antidepressants: 16.8% — 19.9%  Barbiturates: 2.5% — 3.2%  Neuroleptics: 4.9% — 5.8%  Others: 5.5% — 11.3% |
| ^71^Newman & Hassan, 1999 | Canada  1991-92 | C | P  1,659 | Dementia & Depression: DSM-III-R  Cognition: Mini-Mental State Examination (MMSE) | Clinical assessment,  current use | Antidepressants: 3.1 % |
| ^100^Ohayon et al., 1996 | France  1993 | C | P  1,014 | DSM-III-R, DSM-IV,  ICD-10 diagnoses for subjects with sleep problems | Telephone interview,  current and past  consumption | Any psychotropic: 65-74 yrs: 24.3%; >75 yrs: 32.8%  Anxiolytics: 65-74 yrs: 14.2%; >75 yrs: 16.2%  Hypnotics: 65-74 yrs: 5.7 %; >75 yrs: 11.6%  Antidepressants: 65-74 yrs: 2 %; >75 yrs: 2.7% |
| ^94^ Paterniti et al., 1998^4^ | France  1991-93 | C | NP  1,389  (60-70 yrs) | CES-D,  State-Trait Anxiety Inventory (STAI) | Agency interview with prescriptions & containers, regular use past month | Any psychotropic: 21.4%  Anxiolytics/hypnotics: 17.5%  Antidepressants: 1.2%  Neuroleptics: 0.43% |
| ^72^Paterniti et al., 1999^4^ | France  1991-93 | C | NP  1,116  59-71 yrs | CES-D, STAI, MMSE | Agency interview with prescriptions, regular use past month | Any psychotropic: 22.1%  Benzodiazepines: 14.9% |
| ^50^Pérodeau et al., 1992 | Canada  ~ 1989 | C | NP  99  users only | CES-D, Hopkins Symptoms Checklist (HSCL) | Medical records, home interview, past 3 months | Any psychotropic: 100%  Minor tranquillizers: 79%  Antidepressants: 15.6%  Antipsychotics: 3%  Barbiturates: 3% |
| ^79^Pérodeau & Galbaud du Fort, 2000 | Canada  ~ 1997 | C | NP  199 | CES-D, HSCL,  Life events: 2 scales | Home interview with containers, past 3 months | Any psychotropic: 54.7%  - Users: 75% on anxiolytics  11% on antidepressants  5% on antipsychotics  3% on barbiturates |
| ^73^Ried et al., 1998 | USA  1990 | C | P  4,192 | - | HMO prescription claims database | Benzodiazepines: 9% |
| ^117^Santé Québec, 1995 | Canada  1992 | C | P 3,400 | Psychiatric Symptoms Index  (PSI-14) | Home interview, past 2 days | Minor tranquillizers among ≥65 yrs: 21.7% men, 14.7% women |
| ^30^Skood et al., 1993 | Sweden  1986-87 | C | P  494  (85 yr-olds only) | DSM-III-R | Home and outpatient clinic interviews | Any psychotropic: 42.5%  Anxiolytic-sedatives: 34.2%  Antidepressants: 14.0%  Neuroleptics: 5.7% |
| ^32^Stewart, 1994 | USA  1979-92 | L | NP  2,303 | - | Outpatient clinic interview,  past 12 months | Benzodiazepines: 13.0% in 1979, 11.7% in 1992 |
| ^62^Swartz et al., 1991 | USA  1982-83 | C | P  1,839  ≥ 55 yrs | DSM-III-R Diagnostic Interview Schedule (DIS), SCL-90,  Life events scale | Home interview, past 12 months | Benzodiazepines: 14.4% |
| ^118^Tamblyn, 1999 | Canada  1993-1997 | L | Population  93,950 | - | Gov’t prescription claims database | - |
| ^74^Taylor et al., 1998 | United Kingdom  1983-91 | L | P  5,222 | Psychiatric syndromes: GMS-AGECAT | Home interview, past month | Benzodiazepines: 12.8% in 1983, 10.8% in 1991 |
| ^5^Wancata et al., 1997 | Austria  1991-92 | L, 6 months  ­ | N-P  185  ≥ 60 yrs | Mental disorders: Clinical Interview Schedule (CIS) & ICD-9 | Medical records, current use 3 months before admission to nursing home | Any psychotropic: 45.4%  Neuroleptics: 13.0%  Antidepressants: 9.2%  Anxiolytics: 15.7%  Hypnotics: 20.0%  Anticonvulsivants: 2.7%  Others: 4.9% |

**Notes to Table 2:**

1. C = cross-sectional, L = longitudinal

2. P = probability sample, NP = non-probability sample. Unless otherwise noted, all samples include users and non-users of psychotropic drugs. Unless otherwise noted, all participants are ≥ 65 years old.

3. The two reports by Blazer et al. come from the same study.

4. The two reports by Paterniti et al. come from the same study.
