# Supplementary material for: Factors associated with psychotropic drug use among community-dwelling older persons: A review of empirical studies
Source: BMC Nurs. 2004 Aug 13;3:3. doi: 10.1186/1472-6955-3-3 (PMC514897; doi:10.1186/1472-6955-3-3)
Supplement: Additional File 2 — Factors associated with psychotropic drug use among community-dwelling older persons in 32 empirical reports, 1990–2001. Factors associated with psychotropic drug use among community-dwelling older persons. [file 1472-6955-3-3-S2.doc]

###### Table 2

###### Factors associated with psychotropic drug use among community-dwelling older persons in

###### 32 empirical reports, 1990-2001

| **Study** | **Socio-demographic characteristics** | | | | | | | **Life conditions** | | | | | | |
| --- | --- | --- | --- | --- | --- | --- | --- | --- | --- | --- | --- | --- | --- | --- |
|  | **Age^1^** | **Gender** | **Race, Language** | **Marital status** | **SES** | **Health center proximity** | **Insurance** | **Stressful events** | **Illnesses,**  **No. of meds** | **Health perception** | **Social support** | **Mental health** | **Sleep complaints** | **Medical consultations** |
| ^65^Allard et al., 1995 | n.s.^2^  M: I  F: D | 65-74 yrs:  p<.003;  ≥75: n.s. | - | n.s. | Education p<.03  Income p<.01 | - | - | p<.001 | Illnesses p<.001 | - | Social relat. n.s.  Fam. relat. p<.02 | p<.001 | - | - |
| ^66^Antonijoan et al., 1990^3^ | n.s. | p<.02 | - | n.s. | Education n.s. | n.s. | - | - | Illnesses n.s. | n.s. | Social sup. n.s. | n.s. | - | - |
| ^19^Berg & Dellasega, 1996 | - | p<.001 | - | - | - | - | - | - | - | - | - | - | - | - |
| ^6^Blazer et al., 2000 | n.s. | p<.05 | White  p< 0.05 | - | Education & income n.s. | - | n.s. | - | Illnesses n.s. | p<.05 | - | p<.05 | - | p<.01 |
| ^67^Blazer et al., 2000b | T1: n.s.  T2: n.s. | T1: p<.01  T2: n.s. | White T1: p< .02  T2:  p<.0001 | - | Education T1: p<.02, T2: n.s.  Income T1 & T2: n.s. | - | T1: n.s.  T2: n.s. | - | Illnesses T1: n.s.  T2: p<.0007 | T1: p<.0001  T2: n.s. | - | T1: n.s.  T2: n.s. | - | T1: P< .0003  T2: p< .01 |
| ^60^Brown et al., 1995 | n.s. | p<.05 | White p<.05 | n.s. | Education & income n.s. | - | - | - | Illnesses n.s.  Meds n.s. | p<.05 | Living alone n.s. | p<.005 | - | p<.05 |
| ^119^Cans & Rotily,  1991^3^ | - | - | - | - | - | - | - | - | - | - | Living alone  p<.05 | - | - | - |
| ^68^Dealberto et al., 1997, | n.s. I | p<.008 | - | n.s. | Education n.s. | p<.004 | - | - | Illnesses p<.001  Meds p<.003 | - | - | p<.001 | p<.009 | - |
| ^48^Egberts et al., 1997 | p<.05 I | P<.05 | - | - | - | - | - | - | Stroke, arthritis p<.05 ; 7 other illnesses n.s.;  Meds p<.05 | - | - | - | - | p<.05 |
| ^63^Gleason et al., 1998 | n.s. D | p<.01 | White p<.05 | n.s. | Education p<.01 | - | - | - | Illnesses p<.01 | p<.01 | Social sup. n.s. | p<.01 | p<.01 | - |
| ^42^Graham et al., 1998^3^ | p<.05  M: I  F: I | ≤75 yrs: p<.01  ≥75: p<.01 | - | - | - | - | - | - | Meds p<.001 | - | - | - | - | - |
| ^69^Gustafsson et al., 1996 | n.s.  M: D  F: I | 75-79 yrs: n.s.  80-84: n.s.  85-89: p<.001  ≥90: n.s. | - | p<.001 | Occupation p<.001 | - | - | - | Illnesses p<.001 | p<.001 | Social netw. n.s. Loneliness p<.001 | p<.001 | p<.001 | p<.001 |
| ^55^Jorm et al., 2000 | n.s. | p<.05 | - | Divorced p<.05 | Education n.s. | - | - | - | Illnesses n.s. | - | - | p<.05 | p<.05 | p<.05 |
| ^53^Kirby et al., 1999^3^ | n.s. I | p<.0001 | - | - | - | - | - | - | - | - | - | p<.0001 | - | - |
| ^70^Larose, 1996 | n.s.  M: S  F: I | p<.01 | - | n.s. | Income p<.05 | p<.001 | - | - | - | p<.000 | Fam. sup. n.s. | p<.001 | - | - |
| ^49^Mamdani et al., 1999 | p<.001 I | p<.001 | - | - | - | - | - | - | - | - | - | - | - | - |
| ^61^Mayer-Oakes et al., 1993 | n.s. S | n.s. | White p<0.001 | Single p<.05 | Education p<.05  Income n.s. | - | n.s. | - | Meds p<.001 | p<.05 | Social isolat. n.s. | p<.001 | P<.001 | - |
| ^64^McNutt et al., 1994^3^ | n.s. D | p<.001 | White p<0.001 | Single p<.01 | - | - | p<0.05 | - | - | - | - | - | - | - |
| ^71^Newman & Hassan, 1999 | - | p<.05 | - | - | - | p<.05 | - | - | Illnesses p<.01  Meds n.s. | - | - | n.s. | - | - |
| ^100^Ohayon et al., 1996^3^ | p<.05 I | - | - | - | - | - | - | - | - | - | - | - | - | - |
| ^94^ Paterniti et al., 1998 | n.s. I | - | - | - | F: p<.001, M: N.S education  F: p<.006,  M: p<.001 income | - | - | - | F: illnesses n.s.  M: illnesses *less* likely p<.001 | - | - | Depression & anxiety p<.05 | - | - |
| ^72^Paterniti et al. 1999 | - | p<.001 | - | - | - | - | - | - | - | - | - | Depression & anxiety p<.001 | - | - |
| ^50^Pérodeau et al., 1992 | p<.01  D | n.s. | n.s. | - | - | - | - | p<.05 | Illnesses n.s. | - | Social sup. n.s. | n.s. | - | - |
| ^79^Pérodeau & Galbaud du Fort, 2000 | p<.005  D | n.s. | French p<0.005 | n.s. | Education n.s.  Occupation p<.02 | - | - | n.s. | - | - | Social sup. n.s.  Loneliness p<.001 | Depression & anxiety p<.001 | - | - |
| ^73^Ried et al., 1998 | - | p<.02 | - | - | - | - | - | - | Illnesses p<.001 | - | - | - | - | - |
| ^117^Santé Québec, 1995 | - | p<.05 | - | - | - | - | - | - | - | - | - | - | - | - |
| ^30^Skoog et al., 1993^3^ | - | - | - | - | - | p<.05 | - | - | - | - | - | - | - | - |
| ^32^Stewart, 1994^3^ | - | - | - | - | - | p<0.001 | - | - | - | - | - | - | - | - |
| ^62^Swartz et al., 1991 | p<0.05 D | - | - | - | - | - | - | - | - | - | - | - | - | - |
| ^118^Tamblyn, 1999 | - | - | - | - | - | - | p<0.05 | - | - | - | - | - | - | - |
| ^74^Taylor et al., 1998 | p<0.05  Hypno.: I  Anxiol.: S | p<0.05 | - | - | - | - | - | - | - | - | - | p<.05 | - | - |
| ^5^Wancata et al., 1997 | **-** | **-** | **-** | **-** | **-** | p<0.05 | - | **-** | - | - | - | - | - | - |
| **Totals** | S:8 (36%)  N.S:14 (64%)  I:13 (57%)  D: 8 (35%)  S: 2 (8%) | S: 22 (73%)  N.S.: 8 (27%) | Race:  S: 7 (100%)  Language:  S: 1 (50%)  N.S: 1 (50%) | S: 4  (36 %)  N.S.: 7  (64 %) | Education:  S: 7 (54%)  N.S.: 6 (46%)  Income:  S: 4 (44%), N.S.: 5 (56%)  Occupation: S: 2 (100%) | S: 6  (86%)  N.S.: 1  (14%) | S: 2 (33.3%)  N.S.: 4  (66.6%) | S: 2  (66.6%)  N.S.: 1  (33.3%) | Illnesses:  S: 10 (41.6%)  N.S.: 14 (68.4%)  Medications:  S: 4 (66.6%)  N.S.: 2 (33.3 %) | S: 7 (77.7%)  N.S.: 2 (22.3%) | S: 9 (69.2%)  N.S.: 4 (30.8%) | S: 17 (77%)  N.S.: 5  (23%) | S: 5 (100%) | S: 7 (100%) |

Notes to Table 1:

1. In the age column, I: increase; D: decrease; S: stable

2. n.s.: Non statistically significant, p>.05

3. Bivariate analyses only

- : Not studied

M: Male; F: Female
